# Tobacco-specific and combustion pollutants in settled house dust in Malta

**DOI:** 10.20517/jeea.2021.09

**Published:** 2022-02-17

**Authors:** Noel J. Aquilina, Christopher M. Havel, Neal L. Benowitz, Peyton Jacob

**Affiliations:** 1 Department of Chemistry, University of Malta, Msida MSD 2080, Malta; 2 Program in Clinical Pharmacology, Division of Cardiology, Department of Medicine, University of California, San Francisco, CA 94143, USA

**Keywords:** Exposure, Malta, nicotine, polycyclic aromatic hydrocarbons, settled house dust, tobacco-specific nitrosamines

## Abstract

**Aim:**

Most of the carcinogenic pollutants coming from tobacco smoking or other combustion processes tend to accumulate in settled house dust (SHD) over time. This study evaluated the load of these pollutants in smokers and non-smokers’ houses from relatively fresh SHD collected in five different districts on the island of Malta.

**Methods:**

An improved, efficient extraction method to obtain three fractions from a 200 mg of SHD was developed. It was validated for the analysis of nicotine and polycyclic aromatic hydrocarbons (PAH) by GC-MS/MS and nicotelline and TSNA by LC-MS/MS. Kruskal-Wallis H tests were used to evaluate differences across districts, while a Mann-Whitney *U* test was used to check differences between smokers and non-smokers’ houses. Diagnostic ratios were used to evaluate the carcinogenicity of PAH in SHD in Malta.

**Results:**

For all analytes, no statistical difference was observed across different districts, but, in smokers’ houses, 97.9% of the total concentration of all target analytes found in SHD is nicotine, 0.1% is TSNA, and 2.0% is PAH. In non-smokers’ houses, nicotine represents 16.8% of the load, while 0.4% and 82.8% are TSNA and PAH, respectively. The carcinogenicity of the PAH mixture in Maltese SHD, expressed as the mean benzo(a)pyrene equivalent (BaP_eq_) is 371 ng/g.

**Conclusion:**

Indoor activities, ventilation practices, and infiltration of outdoor pollutants contribute to a complex SHD composition. Although the BaP_eq_ is on the lower end of carcinogenicity, the effects of a mixture including tobacco-related potent carcinogens in SHD are largely unknown. In view of indoor, continuous exposure to SHD through several pathways, further research is warranted.

## INTRODUCTION

In any house where tobacco smoking occurs indoors, smouldering fumes from the burning tip of the cigarette, exhaled smoke, and ash are most likely to be the dominant source of particulate matter (PM)^[1–3]^. In non-smoking houses, cooking, space heating using different fuels, candle and incense burning, and other combustion processes are the principal sources of PM^[4–8]^. In both scenarios, the indoor PM concentrations is modulated by infiltration of outdoor generated PM and controlled by the ventilation and filtration conditions within the house. After generation, one expects that airborne PM of different size fractions settle at different rates and hence termed settled house dust (SHD). Different climates and material availability lead to a wide range of building practices. This means that the indoor thermal comfort is generally dictated by the climate and lifestyle. The use of carpeted, heated, wooden, or tiled floors and their cleaning frequency are expected to influence the settling, resuspension, and aging of SHD, as well as its reactivity and dynamics in the indoor environment.

The best indicator of cigarette smoke in the indoor environment has been historically and primarily nicotine^[9,10]^; however, as a semi-volatile organic compound whose vapor pressure ranges from 10^−2^ to to 10^−8^ kPA, it will adsorb to most surfaces, depending on the surface area and air exchange rates, including on airborne PM^[9,11]^. It is thus expected that in smokers’ houses airborne nicotine concentration decreases with time^[9,12–14]^. A minor tobacco alkaloid, nicotelline, has been shown to be a useful marker for the PM derived from tobacco smoke in airborne PM and hence SHD^[15,16]^. The presence of nicotelline is generally indicative of the load of the particle-phase, tobacco-specific derived pollutants in SHD. Sleiman *et al*.^[17]^ showed that, under the right atmospheric conditions, nicotine will contribute to further formation of tobacco-specific nitrosamines (TSNA) over time. TSNA are found in SHD^[18,19]^, but, from an exposure perspective, 4-(methylnitrosamino)-1-(3-pyridyl)-1-butanone (NNK) and N′-nitrosonornicotine (NNN) are particularly important to monitor as they are mutagenic *in vitro* and exhibit carcinogenic activity in laboratory rodents^[20]^. The International Agency for Research on Cancer (IARC) has classified them as carcinogenic in animals and humans (Group 1)^[21,22]^. N′-nitrosoanatabine (NAB) and N′-nitrosoanatalline (NAT) show limited or no mutagenic potential *in vitro* and no carcinogenic activity in laboratory animals (Class 3). Ischemic heart disease and asthma in adults, lower respiratory infections in children younger than five years, and asthma in children^[23]^ indicate there is no risk-free level of exposure to tobacco-related pollutants, not to mention the adverse health effects due to the long-term exposure to secondhand smoke such as heart disease, lung cancer, and stroke^[24]^.

One of the most important pollutant groups linked to the abovementioned typical combustion processes occurring in homes are polycyclic aromatic hydrocarbons (PAH). Of several known PAH, sixteen have been designated as high priority pollutants by the United States Environmental Protection Agency. These PAH are of environmental concern because of their potential toxicity in humans and other organisms and their prevalence and persistence in the environment. Several PAH are probable or known carcinogens such as benzo[a]pyrene (BaP)^[25]^, are generally present in the particle-phase, and hence should be easily detected in SHD. Due to a wide variety of natural and anthropogenic sources, different PAH are generated during combustion processes occurring at various temperature ranges. The use of PAH diagnostic ratios has become a commonly used tool to gather information on their probable emission sources^[26]^. Although it is suggested to use these ratios with caution due to the PAH partitioning across the gaseous and particle-phases, mainly due to meteorological conditions, indoor SHD is probably a more stable matrix to observe compared to outdoor settled dust. The particle-phase PAH are known to be more carcinogenic^[21,27,28]^, but both (2-3 ring) low molecular weight (LMW) PAH and (4-6 ring) high molecular weight (HMW) PAH contribute to the overall carcinogenicity of the PAH mixture expressed as BaP equivalents (BaP_eq_).

Malta has a typical Mediterranean climate. Due to its mild weather in winter and very hot summers, it is customary to have houses built with tiled floors. As precipitation is generally restricted to the months of September-October and January-March, the indoor environment tends to be characterized by the ubiquitous presence of SHD because the opening of windows for natural convection is the most common practice throughout the year, followed by the use of air conditioning systems in the hotter months. Rapid accumulation of SHD generally leads tenants to clean the house frequently, about every three days by dry sweeping, using a vacuum cleaner, or wet dusting the floors. As the adult tobacco smoke prevalence in Malta is still high (20%)^[29]^ and due to the abovementioned lifestyle characteristics, the target pollutants studied are either tobacco smoke specific or linked to any other combustion processes. SHD can easily enter the body by inhalation of resuspended SHD or through ingestion, and the cancer risks associated with a mixture of carcinogenic pollutants or any other healthy effects due to this exposure are largely unknown.

The aim of this study was to improve a method to extract and analyze tobacco- and combustion-related pollutants from SHD. The objectives were: (1) to validate this method using SHD collected from different districts in the island of Malta; (2) to evaluate the levels of a suite of carcinogenic pollutants in SHD collected from tiled floors in smokers and non-smokers’ houses; and (3) to present an evaluation of the carcinogenicity of indoor SHD based on PAH diagnostic ratios.

## EXPERIMENTAL

### Sampling locations

The archipelago of Malta, in the center of the Mediterranean Sea, is made up of six islands, Malta and Gozo being the only two which are inhabited. For logistical reasons, SHD was collected on the bigger island of Malta only, from five districts, whose conurbations are associated with urban, urban background, rural, harbor, and quarries/industrial areas. Further details about these districts and typical activities are given in Supplementary Table 1.

### SHD collection protocol

The sampling campaign was carried out between March and August 2016. The participants were asked to avoid dry sweeping or wet dusting for at least three days before the collection was carried out by a research officer, generally in the evening to allow the tenants to return from work. If possible, SHD was collected from the living room using a brush and a spade. Wherever possible, an outdoor sample was also provided (either from the roof or the yard). In all instances, the collected material was placed in a labeled zip lock bag and transported to the laboratory. The samples were sieved with a 150 μm sieve that was pre-cleaned with methanol and stored in labeled amber bottles at −20 °C prior to analysis.

### Tobacco smoke and combustion related pollutants

Nicotine, nicotelline, and TSNA (NNK, NNN, NAB, and NAT) were extracted and considered indicative of tobacco smoking-specific pollutants. The considered particle-phase PAH, representative of various combustion processes, were phenanthrene (Ph), anthracene (An), fluoranthene (Fluo), pyrene (Pyr), benzo[a]anthracene (BaA), chrysene (Chry), benzo[b]fluoranthene (BbF), benzo[a]pyrene (BaP), indeno[1,2,3-c,d]pyrene (IndP), dibenz[a,h]anthracene (DahA), and benzo[g,h,i]perylene (BghiP).

### Analyses of target pollutants

#### Reagents and standards

Water (H_2_O), methanol (MeOH), dichloromethane (DCM), pentane (Pent), hexane, isopropyl alcohol (IPA), ethyl acetate (EtAc), acetone, toluene, butanol (HPLC grade), sulfuric acid (H_2_SO_4_), hydrochloric acid (HCl), ammonium hydroxide (NH_4_OH), potassium carbonate (K_2_CO_3_), tetrasodium EDTA (Na_4_EDTA), and ammonium formate (AmFor) (reagent grade) were purchased from Fisher Scientific (USA).

Nicotine base was purchased from Sigma Aldrich (USA). Native nicotine and nicotine-d_4_ salts were synthesized as described in Ref.^[30]^. Nicotelline and deuterated nicotelline-d_8_ were synthesized as previously reported^[16]^. Deuterated nitrosamines NNK-d_4_, NNN-d_4_, NAT-d_4_, and NAB-d_4_ and native nitrosamines NNK, NNN, NAT, and NAB were purchased from Toronto Research Chemicals (Canada). The native PAH QTM mix and chrysene-d_12_, were purchased from Supelco (USA) and SPEXCertiPrep (USA), respectively. Deuterated PAH were purchased from Chemservice (USA). Agilent Bond Elut 10 mL and 1 mg silica column were purchased from Agilent (USA).

#### Extraction, cleaning and concentration

**Figure 1.**
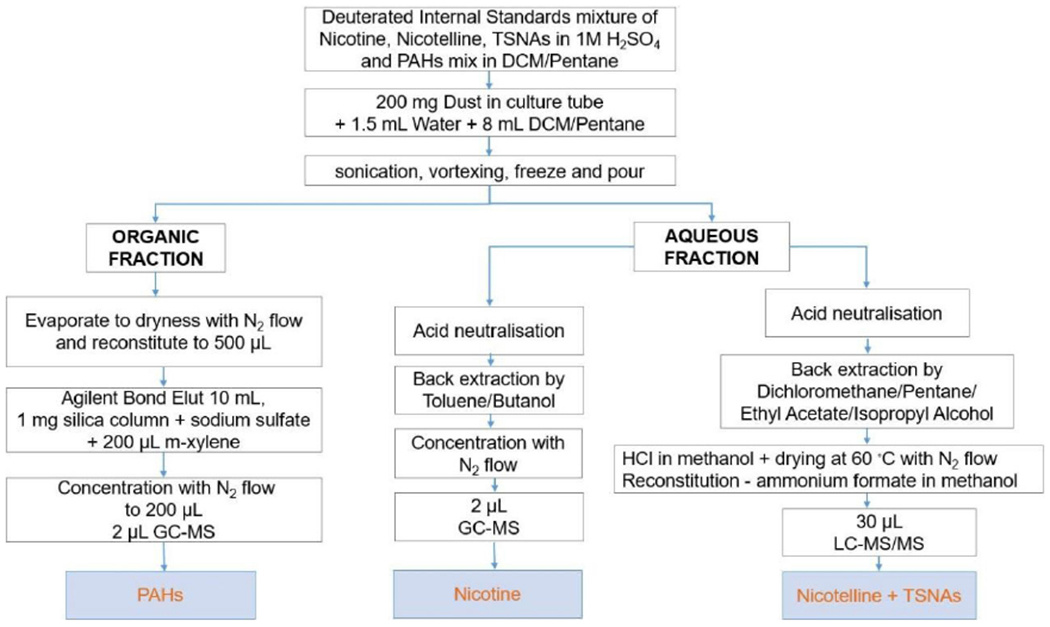
Schematic diagram of the extraction, extract cleanup, and concentration method for the analyses of nicotine, nicotelline, TSNA, and PAH. TSNA: Tobacco-specific nitrosamines; PAH: polycyclic aromatic hydrocarbons.

The method presented in this paper developed by Aquilina *et al*.^[15,31]^ with minor modifications in the matrix, solvent used for extraction, and additional analytes, was suitable to prepare three fractions to be analyzed for nicotine and PAH by GC-MS/MS and nicotelline and TSNA by LC-MS/MS from a 200 mg SHD sample. Figure 1 shows the schematic diagram for the extraction, cleaning, and concentration of the target pollutants from the SHD matrix. Further details about the extraction method are given in the Supplementary Materials.

#### Instrumental analyses

A ThermoFisher LC-MS/MS system (Quantiva) was used for the analyses. A twelve-point calibration curve was used for the nicotelline and TSNA analysis. The concentration levels of the standards, which spanned the monitoring range of interest, were typically LOQ-200 ng/g. 30 μL of the sample extract were injected and the instrument method used was the same as published by Aquilina *et al*.^[15]^. A ThermoFisher GC-MS/MS system (TSQ8000) with an Agilent HP-5MS column (30 m, 0.25 mm, 0.25 μm) calibrated using a twelve-point calibration curve was used for nicotine (LOQ-200,000 ng/g) and PAH (LOQ-10,000 ng/g) analyses.

### Carcinogenicity of PAH in SHD

Using the methodology originally outlined by Nisbet and LaGoy^[32]^ and used by Ma and Harrad^[33]^, the carcinogenicity due to the PAH load in the SHD can be calculated in the context of indoor activities and the location from where the SHD was collected. Unfortunately, this calculation could not be extended to the tobacco-related pollutants in addition to PAH in SHD as there are not yet sufficient data about NNK and NNN in this regard.

### Statistical methods

All statistical analysis was carried out with SPSS version 27 (IBM Corp. Released, 2020. IBM SPSS Statistics for Windows, Version 27.0. Armonk, NY: IBM Corp). To identify if there was significant difference in the analytes mean rank across smoking and non-smoking houses, a Mann-Whitney *U* test was performed. A Kruskal-Wallis H test was run to evaluate whether absolute measured values of all the analytes differed across the five districts in Malta. We considered differences between groups to be statistically significant when *P* < 0.05.

## RESULTS

Clay soil, collected several inches beneath the surface during the winter when the soil was moist and subsequently dried in an oven, was used as the blank matrix, because concentrations of the analytes were below the limit of quantitation (LOQ) compared to SHD or urban dust samples. The performance of the analytical method, including precision and accuracy, was determined from the results of analyzing four replicates of 200 mg powdered and dried clay soil samples (blank matrix), spiked with an aqueous solution of specified amounts of nicotelline, nicotine, TSNA, and PAH. The target analytes were extracted from this matrix and analyzed as described in the Experimental Section. The corresponding percentage recovery is summarized in Supplementary Table 2. LOQs were determined as the minimum concentration on the calibration curves [Supplementary Table 2 and 3] that do not exceed an RSD of 20% for replicate analyses of the blank clay samples spiked with analytes. Analyses of the blank clay soil gave results below the LOQs for all analytes.

Table 1 summarizes the target pollutant concentrations of indoor SHD collected in Malta. Apart from IndP, the detection frequency for all target pollutants was over 70%.

**Figure 2.**
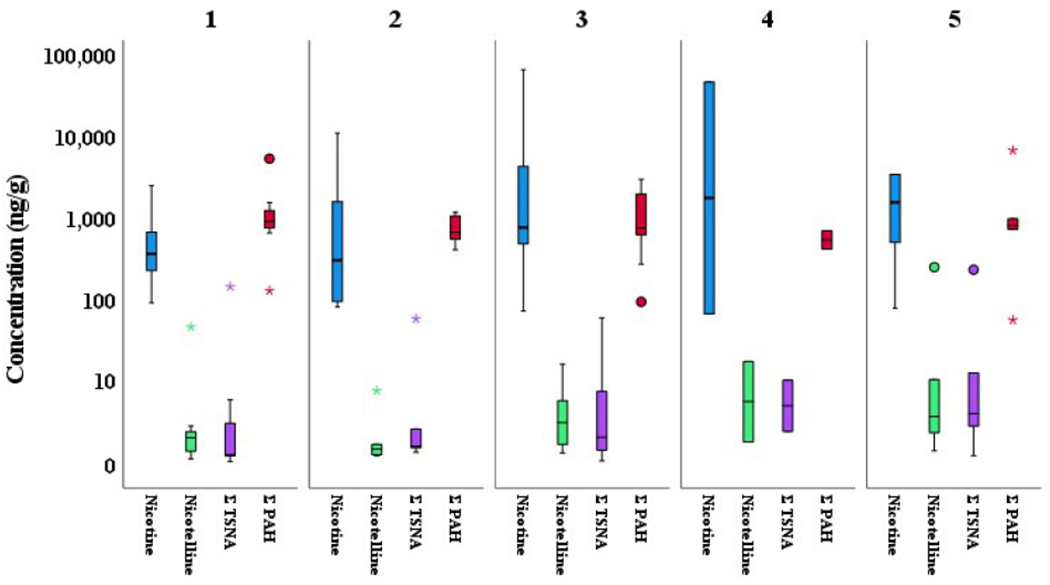
Concentration (in ng/g) of nicotine, nicotelline, ΣTSNA, and ΣPAH in indoor SHD in different districts. District classification (number of samples): 1: urban (10); 2: urban background/rural (7); 3: urban/harbor (14); 4: urban background/rural (7); 5: quarries/industrial/trans-shipment hub (16). ◯: Outliers; *: extreme values.

The variability of all pollutants in SHD across the different Maltese districts (1-5) is illustrated in Figure 2. Reference is made to Supplementary Table 1 and corresponding activities across the different districts in Malta to help explain the variability in pollutants. Although there is more variability in the nicotine concentration, the results of the Kruskal-Wallis H test in Supplementary Table 4 indicate that there was no statistical difference in the concentration of all target pollutants [H(4) = ranges from 0.933 to 5.579, all *P* > 0.233] across the districts.

**Figure 3.**
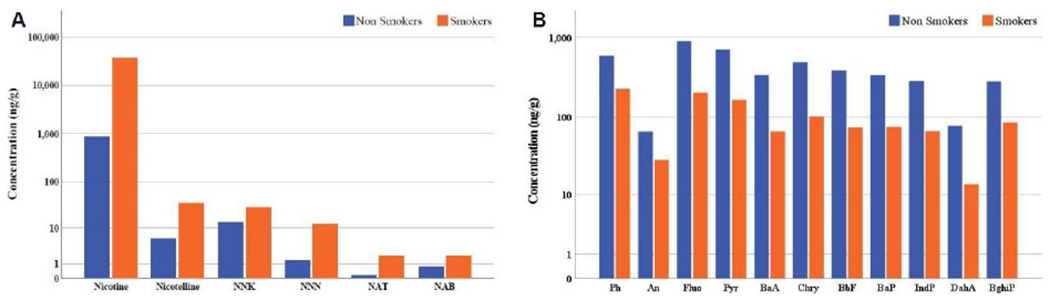
Concentration (in ng/g) in indoor SHD in smokers and non-smokers’ houses of: (A) nicotine, nicotelline, and TSNA; and (B) PAH. SHD: Settled house dust; TSNA: tobacco-specific nitrosamines; PAH: polycyclic aromatic hydrocarbons; NNK: 4-(methylnitrosamino)-1-(3-pyridyl)-1-butanone; NNN: N′-nitrosonornicotine; NAT: N′-nitrosoanatalline; NAB: N′-nitrosoanatabine; Ph: phenanthrene; An: anthracene; Fluo: fluoranthene; Pyr: pyrene; BaA: benzo[a]anthracene; Chry: chrysene; BbF: benzo[b]fluoranthene; BaP: benzo[a]pyrene; IndP: indeno[1,2,3-c,d]pyrene; DahA: dibenz[a,h]anthracene; BghiP: benzo[g,h,i]perylene.

Although nicotine is dominant in smokers’ houses (two orders of magnitude higher, as shown in Figure 3A), for the whole indoor dataset, its median concentration is double that of the sum of PAH (ΣPAH) and substantially higher than the sum of TSNA (ΣTSNA). Tobacco smoking-related pollutants were detected in all samples, as confirmed by the marker for PM matter derived from tobacco smoking, nicotelline. As the number of samples for non-smokers is higher, the median level of ΣTSNA is indicative of the levels expected in non-smokers’ SHD. The mean levels of the tobacco-related pollutants for both smokers and non-smokers’ houses measured in this study are compared to those of other studies in Table 2. A Mann-Whitney *U* test was performed [Supplementary Table 5], and the results show that there is a statistical difference between smokers and non-smokers’ houses for tobacco-related pollutants (*P* < 0.001, 2-tailed) and all PAH (*P* < 0.05, 1-tailed). The mean levels of the tobacco-related pollutants in SHD for both smokers and non-smokers’ houses measured in this study are compared to those of a very limited number of studies in Table 2.

All studies in Table 2 show that smokers’ houses have a nicotine concentration which is typically one to several orders of magnitude higher than in non-smoking houses. Where reported, nicotelline concentrations are higher in smokers’ houses. For the study carried out in Spain, the TSNA concentrations are an order higher in both smokers and non-smokers’ houses when compared to the other studies. There is a substantial difference in the concentrations for all components between the two studies in California, but there was no information on specific house characteristics or smoking patterns to explain this difference. The concentrations of nicotine, nicotelline, and TSNA in Malta were similar to the study in California performed in 2015 but much less than the other two studies.

Figure 3B shows a somewhat different characteristic where the individual PAH concentrations were higher for non-smoking houses. A set of outdoor settled dust was collected wherever available, and the statistics for all pollutants analyzed in this set are reported in Supplementary Table 6.

In this study, the PAH diagnostic ratios outlined in^[26]^ were applied to indoor SHD and outdoor settled dust. Table 3 shows the mean ratios calculated from the PAH available data with a description of the possible PAH sources. It should be noted that the indoor and outdoor diagnostic ratios do not vary substantially.

Ma and Harrad^[33]^ reviewed 35 studies, most of them collecting SHD (of size < 150 μm) with a vacuum cleaner and analyzed for PAH. The calculation of the BaP_eq_ for the PAH mixture in SHD was based on the methodology outlined by Nisbet and LaGoy^[32]^. Five of these studies looked into the ΣPAH in indoor SHD that was collected by hand brushing, as was the case in Malta. Table 4 compares their findings.

From the Spearman-Rho correlation table, for all pollutants in the SHD shown in Supplementary Table 7, while nicotine correlates in a statistically significant manner with nicotelline and all TSNA (*P* < 0.01, two-tailed), it shows a negative correlation with all PAH. The relationship is significant (*P* < 0.05, two-tailed) with BaA, Chry, BbF, and DahA. Nicotelline and all TSNA show a non-significant, negative correlation with any of the PAH as well as ΣPAH.

Supplementary Table 8 compares the load (ng/g of analyte compared to the total analytes extracted from SHD in ng/g, expressed as a percent) of nicotine, ΣTSNA, and ΣPAH in indoor and outdoor SHD and not distinguishing between smokers and non-smokers’ houses. The indoor SHD has a high content of nicotine (85.1%) and only 14.8% of ΣPAH and 0.1% of ΣTSNA when compared to the outdoor SHD.

The load of the same pollutants for indoor SHD in smoking and non-smoking houses was also compared. In this case, for smokers’ houses, the load of the targeted pollutants in SHD is dominated by nicotine (97.9%), while ΣPAH and ΣTSNA represent only 2% and 0.1%, respectively. For non-smokers’ houses, the major contribution comes from ΣPAH (82.8%), while nicotine and ΣTSNA represent 17.2% of the load in SHD.

## DISCUSSION

A validated method was devised to extract aqueous and organic fractions from SHD and clean this typically complex matrix to allow analyses of a suite of pollutants using both GC-MS/MS and LC-MS/MS. The previously published method by the authors for the extraction and analyses of the same pollutants from particulate matter on filters^[15,31]^ was adapted and validated for SHD. It was more flexible in analyzing a wider range of analytes from a single 200 mg sample of SHD and less time consuming in weighing a single rather than three samples for three separate analyses. It was shown that the analytical method was reliable given the amount of SHD required.

For the whole indoor dataset, the median concentration of nicotine was double that of ΣPAH and substantially higher than the ΣTSNA. This is not unexpected given that nitrosamines in the atmosphere are in much lower abundance than other less reactive organic pollutants^[40]^. Comparing the maximum and median values of nicotine, nicotelline, and ΣTSNA, it is clear that smokers’ houses skew the load of tobacco-related pollutants in SHD when compared to non-smoking houses. The detection of nicotelline in all SHD samples is indicative of either tobacco smoke-derived PM generation indoors, as is the case of smokers’ houses, or of infiltration of tobacco smoke-contaminated PM, as reported by Aquilina *et al*.^[15,41]^. Analysis of the tobacco-related components in SHD in the different districts of Malta indicated more variability in nicotine levels because certain districts (3-5) are known to have a higher tobacco smoke prevalence.

Comparing this study with the limited number of studies involving nicotine and TSNA in SHD, the study in California, in 2015^[18]^, showed similar tobacco-component concentrations to those obtained in Malta. Malta has a higher tobacco prevalence, which could explain why the levels of nicotelline and TSNA are surprisingly high in relatively fresh SHD, when compared to the SHD collected from carpets in California. Airborne nicotine in secondhand smoke is more likely to condense on PM before the latter settles. The carpets would serve as a sink for SHD to age, leading to more TSNA formation over time, a process typical of thirdhand smoke^[19,39–41,42–46]^. Nitrosation of nicotine under specific conditions leads to higher TSNA levels^[11]^, more than infiltrated PM contaminated with TSNA^[31]^. The results from the study in Spain are substantially higher than the others, possibly because tobacco smoking was frequent and excessive with poor indoor ventilation.

In Malta, in the colder months, the tendency is to keep windows closed and hence only fine PM (< PM_2.5_) manages to infiltrate the houses. Although it is expected that PAH would be higher in smokers’ homes, as smoking-derived PAH would add to the PAH generated from other activities^[47–50]^, this was not the case in this study. Such variability in PAH in smokers and non-smokers’ houses was also reported by Hoh *et al.*^[45]^. It is not unexpected that pollutants pertaining to tobacco smoke (nicotine, nicotelline, and TSNA) do not correlate with combustion pollutants such as PAH. Hoh *et al*.^[45]^ argued that PAH may be transported within the different rooms in a house and that the floor type may affect dynamics/reactivity of SHD or PAH in SHD; as discussed above, indoor activities other than smoking cannot be ignored as important contributors to higher levels of PAH in non-smoking houses.

The levels of PAH in the five districts were of the same order of magnitude. Although the outdoor activities in the districts should be different in accordance with their classification, the area of the island in conjunction with very good air mixing would not lead to specific gradients in outdoor concentrations. In addition, if it is assumed that the indoor sources of ΣPAH are relatively similar across the districts, due to similar lifestyles on such a small island, it would explain why ΣPAH do not show high variability^[51]^; this observation was confirmed by the Kruskal-Wallis H test for all pollutants.

If the indoor SHD is generally composed of a finer fraction, unlike outdoor SHD, it seems that diagnostic ratios are not affected by particle size^[52]^. Although in the indoor environment some activities such as space heating using gas, fireplace use, cooking styles such as grilling, use of incense/scented candles, and tobacco smoking could be important sources of PAH^[47,48,50]^, in Malta, the accumulation of PAH is possibly avoided due to some lifestyle practices. Fireplaces are not common and living rooms’ space heating is typically done with a heating, ventilation, and air-condition unit. Furthermore, although cooking styles might differ in different houses, the general tendency is to have natural ventilation by opening windows and doors all year round, except in the colder months. In this way, after some time, there is probably a steady state of infiltration and exfiltration^[53,54]^. This would lead to the outdoor sources playing an important role in the chemical composition of the indoor SHD and hence explaining why the mean values of the indoor and outdoor diagnostic ratios are similar. Apart from the abovementioned indoor PAH sources, the proximity to trafficked roads of most houses in Malta cannot be ignored, a fact noticeable in Figure 2, where the classification of the different districts does not contribute to substantially differing levels of PAH. This could also explain why more PAH in non-smokers’ homes were noticed [Figure 3B], as windows are possibly opened more in smokers’ homes to ventilate cigarette smoke and reduce its smell. However, as other pollutants such as alkylated PAH were not monitored, the identification of specific sources of PAH in SHD is difficult, possibly also explaining the lack of correlation between tobacco-related components and PAH.

The diagnostic ratios calculated from the PAH levels in SHD give inference on certain activities. The ratio [An/(An + Ph)] values indicate petrogenic emissions, certainly dominated by asphalt production and usage that was substantial in several infrastructural projects in Malta in recent years, but also from cars and trucks dripping fuels and lubricating oils around the streets. The finer fraction of the roadside dust would certainly infiltrate houses given the typically dry climate of Malta with limited scavenging of these PAH by precipitation. The Fluo/(Fluo + Pyr) ratio is associated with grass and wood combustion, certainly not coal combustion. Coal is not used in Malta; however, in the summer months, dried grass is normally burnt in open fields. Wood combustion would be due to the use of fireplaces, although this is not widespread given the country’s climate, as discussed above. The remaining ratios indicate that the main sources of PAH are most likely linked to vehicles/internal combustion engine and other pyrogenic emissions where any organic matter is subjected to high temperatures but with insufficient oxygen for complete combustion.

In a review of several studies on the calculation of the BaP_eq_ obtained in SHD, Ma and Harrad^[33]^ argued that ΣPAH (127-115,817 ng/g) and BaP_eq_ (19-15,530 ng/g) exhibit a lot of variability because of the nature of the different sampling protocols and size fraction of the SHD collected. The overall ΣPAH and BaP_eq_ (mean ± SD) for all the studies were 14,105 ± 4025 and 1897 ± 552 ng/g, respectively. With reference to Table 4, the mean ΣPAH and BaP_eq_ values for this study were very similar to what was obtained in Palermo, a city which is close to Malta and of similar climate and socioeconomic characteristics. If one excludes the results for Shanghai, although the levels in Malta are on the lower end of the concentration scale, the load of PAH in relatively fresh SHD is not to be ignored, given the activities in Malta described above.

The opening of windows as the preferred ventilation method of houses might explain the relatively low load of ΣPAH in SHD. This argumentation might not apply to nicotine due to its characteristic adherence to surfaces^[55]^. Although one expects that the load of nicotine in outdoor SHD to be much lower, for the abovementioned reason, nicotine is easily detected in outdoor air, even though the concentration is an order of magnitude less. In Malta, smoking occurring outside on terraces, balconies, or on the roof of the house is common practice and that could be a contributing factor to a relatively high load of nicotine. For ΣTSNA, the expected load is low compared to other pollutants in outdoor SHD. This is not surprising, and, although the occurrence of TSNA is modulated by atmospheric conditions and consequent reactivity/degradation processes, it has been shown that TSNA are found in airborne PM^[31,40]^. For ΣPAH, the situation is somewhat more complex. Although indoor ΣPAH concentrations are expected to be higher due to indoor activities, the load depends immensely on whether tobacco smoking occurs.

When the load for the same pollutants in indoor SHD was evaluated according to the smoking status in the houses, in smokers’ houses, the load of the SHD was dominated by nicotine and ΣPAH represented only 2%, probably because these houses are more ventilated to avoid the smell of cigarette smoke. It has been shown that, in Maltese houses, the air exchange rate has an important effect on the removal of PM^[41]^. For non-smoking houses, the major contribution comes from ΣPAH, yet nicotine and ΣTSNA represent 17.2% of the load in SHD, certainly attributed to infiltration of outdoor, tobacco smoke-contaminated PM.

## CONCLUSIONS

This was the first study of its kind on the island of Malta, in the Mediterranean Sea, reporting both tobacco smoke and other combustion processes pollutants in indoor and outdoor SHD. In a country where the tobacco smoke prevalence is still relatively high and, in some cases, smoking occurring indoors, very few studies have characterized indoor SHD over tiled floors for nicotine as the primary component of secondhand smoke, nicotelline as the marker of tobacco smoke-derived PM, and TSNA, namely the potent carcinogens NNK and NNN.

As the indoor air is generally dependent on combustion activities associated with lifestyle habits and to some extent is influenced by the outdoor air due to ventilation being driven by natural convection, SHD was also analyzed for combustion pollutants, namely PAH, where the higher molecular weight particle-phase components are known to be carcinogenic. Although the mean BaP_eq_ of the Maltese SHD is on the lower end of carcinogenicity compared to other studies, the effects of a mixture of potent carcinogens in SHD that is relatively fresh are largely unknown. This is worrying in relation to a continuous human exposure via the inhalation, ingestion, and dermal sorption pathways. The mass fraction of the different pollutants in indoor and outdoor SHD gives information not only on the possible sources but also on the possible concentration of pollutants in other rooms based on air exchange rates, removal rates, and other dynamic considerations in multi-zone houses.

The main limitation of the study could be the relatively small number of samples used and the lack of information on the indoor activities to gauge the variability of the pollutants, as well as link the determinants that influence the personal exposure to the suite of pollutants studied. Future research would require more samples collected from different rooms, identifying different furnishings and floor types, time-activity information, ideally supplemented with ventilation information within the house. Although at times such information is challenging to acquire, nowadays with Internet of Things infrastructure such acquisition could be more achievable. The development of artificial intelligence algorithms to identify the most important predictors would allow policy makers engaged in public health to possibly predict exposure to a mixture of carcinogenic pollutants derived from different sources and calculate the associated cancer risk. To date, risk calculations associated due to such mixtures are still a very challenging feat.

In light of the current pandemic, where a hybrid mode of working is desirable, more people are present at home for a longer time. The exposure to SHD would be expected to be higher as various indoor activities are quite intrinsically distinct from office microenvironments. On the other hand, movement and social activity restrictions might induce smokers to smoke more frequently and possibly in home spaces without adequate ventilation. In both scenarios, SHD levels would possibly increase and the implications on enhanced exposure to the abovementioned suite of pollutants are worth investigating further.

## Supplementary Material

Aquilina_Tob combustion pollutans house dust Malta_JEEA 2022 Supporting Info

## Figures and Tables

**Table 1.** Descriptive statistics for all pollutants (in ng/g) of indoor SHD collected in Malta

Pollutant	Valid *n*	Detection frequency (%)	Mean	SD	Min	Max (ng/g)	Q1	Q2	Q3
**Nicotine**	54	100	18,246	43,260	69	188,715	404	1618	11,164
**Nicotelline**	54	100	21	47	BLOQ	258	1	3	17
**NNK**	54	100	19	44	BLOQ	216	1	3	12
**NNN**	54	100	5	10	BLOQ	38	BLOQ	1	4
**NAT**	54	100	1	2	BLOQ	8	BLOQ	BLOQ	1
**NAB**	54	100	1	1	BLOQ	5	BLOQ	1	1
**Ph**	44	81	435	767	15	4295	87	161	328
**An**	44	81	49	116	1	701	5	10	24
**Fluo**	39	72	692	1486	32	8270	88	146	392
**Pyr**	39	72	542	1089	14	6011	84	134	431
**BaA**	42	78	243	567	3	3299	20	39	191
**Chry**	41	76	362	683	12	3771	68	126	300
**BbF**	39	72	296	609	BLOQ	3318	45	70	232
**BaP**	40	74	266	571	BLOQ	3200	21	44	244
**IndP**	37	69	232	453	BLOQ	2433	32	52	223
**DahA**	43	80	55	113	BLOQ	537	6	12	48
**BghiP**	44	81	201	381	3	2087	35	66	141
**ΣPAH**	45	83	3169	6702	3	39,167	441	818	2198
**ΣTSNA**	54	100	26	54	BLOQ	267	2	5	18

Q1, Q2, and Q3 are the 25th, 50th, and 75th percentiles, respectively. ΣPAH: Sum of PAH; ΣTSNA: sum of TSNA; BLOQ: below limit of quantitation.

**Table 2.** Comparing tobacco-related pollutants (in ng/g) in indoor SHD with other studies

House	Floor type	*n*	Nicotine	Nicotelline	NNK	NNN	NAB	NAT	Place country, year Ref.
**S**	Carpet	2	98,200^a^	173^a^	84^a^	35.4^a^	-	-	California USA, 2013 [16]
**NS**		5	1990^a^	2.7^a^	3.2^a^	1.27^a^	-	-	
**S**	Tiled	22	26,000	-	540	20	510	70	Tarragona Spain, 2014 [34]
**NS**		24	2300	-	40	4	0	10	
**S**	Carpet	6	7000	8.0	3.7	1.6	< 0.2	< 4.2	California USA, 2015 [18]
**NS**		20	520	1.0	< 0.5	< 1.4	< 0.2	< 4.2	
**S**		6	7800	7.1	5.8	2.9	0.2	< 4.2	
**NS**		20	510	0.6	0.5	< 1.4	< 0.2	< 4.2	
**S**	Tiled	16	11,164	15.9	9.4	5.7	1.9	1.3	This study Malta, 2016
**NS**		38	418	2.2	1.6	0.2	0.5	0.1	

a This study reported only mean concentrations. The other studies reported median concentrations.

S: Smokers’ houses; NS: non-smokers’ houses; SHD: Settled house dust; NNK: 4-(methylnitrosamino)-1-(3-pyridyl)-1-butanone; NNN: N′-nitrosonornicotine; NAT: N′-nitrosoanatalline; NAB: N′-nitrosoanatabine.

**Table 3.** PAH diagnostic ratios calculated for indoor (*n* = 45) and outdoor (*n* = 20) SHD (in ng/g)

Mean ratio	Indoor	Outdoor	Description
**An/(An + Ph)**	0.09	0.10	Petrogenic emissions (< 0.1)
**Fluo/(Fluo + Pyr)**	0.52	0.58	Grass, wood, coal combustion (> 0.5)
**BaA/(BaA + Chry)**	0.32	0.34	Coal combustion (0.2-0.35); vehicle emissions (> 0.35)
**IndP/(IndP + BghiP)**	0.44	0.44	Petroleum combustion (0.2-0.5)
**BaP/BghiP**	0.92	0.85	Traffic emissions (> 0.6)
**Σ LMW/Σ HMW**	0.39	0.20	Pyrogenic (< 1)

SHD: Settled house dust; PAH: polycyclic aromatic hydrocarbons; Ph: phenanthrene; An: anthracene; Fluo: fluoranthene; Pyr: pyrene; BaA: benzo[a]anthracene; Chry: chrysene; BaP: benzo[a]pyrene; IndP: indeno[1,2,3-c,d]pyrene; BghiP: benzo[g,h,i]perylene; Σ LMW: low molecular weight (2-3 ring PAH); Σ HMW: high molecular weight (4-6 ring PAH).

**Table 4.** Comparing ΣPAH and BaP_eq_ (both in ng/g) in indoor SHD with other studies

Place	Year	*n*	ΣPAH	BaP_eq_	Ref.
**Shanghai, China**	2005	25	20,674	4393	[35]
**Palermo, Italy**	2006	45	5111	262	[36]
**Triunfo, Brazil**	2008	9	4091	288	[37]
**Delta State, Nigeria**	2009	30	127	NR	[38]
**Shanghai, China**	2010	22	11,575	829	[39]
**Malta**	2016	45	3172	371	**This Study**

NR: Not reported.

## Data Availability

Not applicable.
