# Supplementary material for: Tobacco-specific and combustion pollutants in settled house dust in Malta
Source: J Environ Expo Assess. Author manuscript; Available in PMC 2022 Apr 30. (PMC9056077; doi:10.20517/jeea.2021.09)
Supplement: Aquilina_Tob combustion pollutans house dust Malta_JEEA 2022 Supporting Info [file NIHMS1793452-supplement-Aquilina_Tob_combustion_pollutans_house_dust_Malta_JEEA_2022_Supporting_Info.pdf]

DOI: 10.20517/jeea.xxxx.xx
Journal of Environmental Exposure Assessment

Supplementary Material: tobacco-specific and combustion pollutants in settled house dust in Malta

Details of the extraction, cleaning and concentration of PAHs, Nicotine, Nicotelline and TSNAs from settled house dust (SHD).

Extraction of PAHs

Approximately 200 mg of sieved SHD were weighed and placed in a 16 × 125 culture tube. 50 μL of Nicotine-d_4 internal standard and 50 μL of Nicotelline + TSNA internal standard mix were prepared in advance in 0.5 mL 1M H_2SO_4 per sample. Similarly, 50 μL of PAH internal standard mix was prepared in 0.5 mL DCM/Pentane (50:50) per sample. Each dust sample was spiked with the abovementioned internal standard mixes and 1.5 mL of water and 5 mL of DCM/Pentane(50:50) were added. The mix was vortexed for 30 s and sonicated for 30 min at 25 °C. After vortexing for 30 s, the tubes were centrifuged at 3700 rpm for 4 min and then frozen in an acetone-dry ice bath.

The organic phase (this is Fraction 1) was transferred in a 13 × 100 tube and kept for PAHs analysis.

Fraction 1: PAHs

This fraction was blown to dryness at 25 °C under a gentle stream of nitrogen and reconstituted with 500 μL of hexane. 300 mg of anhydrous sodium sulfate as a drying agent were added to an Agilent Bond Elut 10 mL, 1 mg silica column. The silica column was prewashed with 3 mL MeOH followed by 3 mL acetone, conditioned with 3mL hexane/DCM (50:50) followed by 6 mL hexane, loaded with 500 μL of reconstituted extract in hexane and eluted with 6 mL of hexane/DCM (70:30) in a 13 × 100 tube. 200 μL of m-xylene were spiked in the collected eluent and blown down under a gentle stream of nitrogen to a final extract volume of 200 μL which was

© The Author(s) 2021. Open Access This article is licensed under a Creative Commons Attribution 4.0 International License (https://creativecommons.org/licenses/by/4.0/), which permits unrestricted use, sharing, adaptation, distribution and reproduction in any medium or format, for any purpose, even commercially, as long as you give appropriate credit to the original author(s) and the source, provide a link to the Creative Commons license, and indicate if changes were made.

transferred with a glass Pasteur pipette in a GC vial with a 250 μL insert. 2 μL were injected in a GC-MS.

Extraction of Nicotelline + TSNAs

After Fraction 1 was removed, 1 mL of 45% potassium carbonate + 5% Na_4EDTA were added to the remaining aqueous phase followed by 8 mL of DCM/Pentane/EtOAc/IPA (40:40:15:5). After vortexing for 1 min and centrifuging at 3700 rpm for 4 min, the two phases were placed in an acetone-dry ice bath.

At this stage the organic phase was split as follows:

Fraction 2: In a 13 × 100 tube containing 0.5 mL of 1M H_2SO_4, 3 mL of the organic phase were poured, for **Nicotine** to be extracted from it.

Fraction 3: In another 13 × 100 tube containing 0.5 mL of 1M H_2SO_4, the remaining 5 mL of the organic phase were poured, for **Nicotelline + TSNAs** to be extracted from it.

Both Fractions 2 & 3 were vortexed for 1 min, centrifuged at 3700 rpm for 4 min and frozen in an acetone-dry ice bath after which the organic phase was discarded.

Fraction 2: Nicotine

The remaining aqueous phase was neutralized with 0.5 mL 50% K_2CO_3/2N NH_4OH and 0.5 mL 90:10 Toluene/Butanol were added. The two phases were vortexed for 1 min, centrifuged at 3700 rpm for 3 min and frozen in an acetone-dry ice bath. The 0.5 mL organic phase was poured in an EDTA deactivated GC vial and injected 2 μL in GC-MS.

Fraction 3: Nicotelline + TSNAs

The remaining aqueous phase was neutralized with 0.5 mL 45% K_2CO_3/5% Na_4EDTA and 5 mL 40:40:15:5 DCM/Pent/EtAc/IPA were added. The mixture was vortexed for 1 min, centrifuged at 3700 rpm for 3 min and frozen in an acetone-dry ice bath. The organic phase was poured in a 13 × 100 tube and spiked with 100 μL of 1% HCl in MeOH and dried at 60 °C under a gentle nitrogen flow. Reconstitution of analytes was done with 200 μL 0.1 M ammonium formate in 20% MeOH. 30 μL were injected in

LC-MS/MS.

Supplementary Table 1. Characteristics and statistics of districts in Malta

District	Name	Population	Area[a] (%)	Type
1	Northern Harbour	131,056	7.6	Urban
2	Northern	69,467	23.3	Urban background/Rural
3	Southern Harbour	80,170	8.3	Urban/Harbour
4	Western	59,817	22.9	Urban background/Rural
5	South Eastern	68,044	17.2	Quarries/industrial/ Trans-shipment hub

[a]Percentage area occupied by district in relation to the surface area of Malta.

Supplementary Table 2. Precision and accuracy for determination of nicotine, nicotelline, TSNAs and PAHs in analyte spiked clay soil[a] used as QC samples

Pollutant	LOQ (ng/g)	Actual (ng/g)	Measured (ng/g)	SD (ng/g)	% RSD	% Accuracy	% Recovery
**Nicotine**	1.4	1000.0	976.9	20.7	2.1	-2.3	97.7
		333.3	351.3	0.5	0.1	5.4	105.4
		111.1	115.2	0.5	0.4	3.7	103.7
		37.0	38.0	0.3	0.7	2.7	102.7
		12.3	12.4	0.0	0.3	0.3	100.3
		4.1	4.3	0.1	1.5	4.8	104.8
		1.4	1.2	0.0	1.6	-14.5	85.5
**Nicotelline**	0.36	100.0	91.4	7.6	8.3	-8.6	91.4
		33.3	32.1	2.6	8.3	-3.7	96.3
		11.1	11.8	1.2	9.9	5.8	105.8

		3.7	4.0	0.2	5.5	8.8	108.8
		1.2	1.3	0.1	6.3	6.4	106.4
		0.4	0.4	0.0	6.2	-4.9	95.1
**NNK**	0.14	100.0	100.3	0.4	0.4	0.3	100.3
		33.3	30.4	3.7	12.3	-8.7	91.3
		11.1	10.9	0.4	3.5	-2.2	97.8
		3.7	3.7	0.2	6.3	-0.2	99.8
		1.2	1.2	0.1	9.4	0.7	100.7
		0.4	0.4	0.0	5.8	3.5	103.5
		0.1	0.1	0.0	9.5	4.2	104.2
**NNN**	0.02	100.0	79.5	18.0	22.6	-20.5	79.5
		33.3	26.9	0.6	2.2	-19.3	80.7
		11.1	10.5	0.8	7.7	-5.4	94.6
		3.7	3.8	0.2	4.8	2.5	102.5
		1.2	1.3	0.0	3.6	3.2	103.2
		0.4	0.4	0.0	7.1	-5.2	94.8
**NAB**	1.23	100.0	92.9	4.9	5.2	-7.1	92.9
		33.3	34.1	0.9	2.6	2.3	102.3
		11.1	11.4	0.7	6.2	2.8	102.8
		3.7	3.9	0.1	2.8	4.9	104.9
		1.2	1.2	0.1	7.1	1.0	101.0
**NAT**	0.02	100.0	100.2	4.2	4.2	0.2	100.2
		33.3	33.3	0.1	0.3	-0.2	99.8
		11.1	11.0	0.3	3.1	-1.2	98.8
		3.7	3.7	0.0	1.0	1.2	101.2
		1.2	1.2	0.0	1.0	0.8	100.8
		0.4	0.4	0.0	2.1	1.9	101.9
		0.1	0.1	0.0	1.2	-2.0	98.0

		0.05	0.0	0.0	0.9	-2.1	97.9
		0.02	0.0	0.0	1.8	-0.2	99.8
**Ph**	0.82	200.0	172.2	23.2	13.5	-13.9	86.1
		66.7	66.1	1.4	2.1	-0.9	99.1
		22.2	22.6	0.9	4.0	1.7	101.7
		7.4	6.8	0.1	0.7	-8.2	91.8
		2.5	2.5	0.2	8.6	0.6	100.6
		0.8	0.9	0.0	4.3	3.7	103.7
**An**	0.09	200.0	179.6	18.1	10.1	-10.2	89.8
		66.7	66.0	1.5	2.3	-0.9	99.1
		22.2	23.5	0.9	4.0	5.7	105.7
		7.4	6.8	0.3	4.6	-7.6	92.4
		2.5	2.4	0.1	5.3	-3.5	96.5
		0.8	0.9	0.0	2.4	8.0	108.0
**Fluo**	2.47	200.0	182.1	13.8	7.6	-8.9	91.1
		66.7	65.6	0.4	0.6	-1.6	98.4
		22.2	23.6	0.2	1.0	6.4	106.4
		7.4	7.1	0.2	2.2	-3.6	96.4
		2.5	2.4	0.1	2.7	-1.2	98.8
**Pyr**	7.41	200.0	186.8	16.8	9.0	-6.6	93.4
		66.7	66.5	4.1	6.2	-0.3	99.7
		22.2	23.9	1.2	4.9	7.5	107.5
		7.4	6.9	0.3	4.6	-6.8	93.2
		2.5	2.5	0.1	2.3	1.7	101.7
**B(a)A**	0.09	200.0	184.5	11.4	6.2	-7.7	92.3
		66.7	64.9	2.0	3.2	-2.7	97.3
		22.2	23.9	0.4	1.7	7.4	107.4
		7.4	6.8	0.2	3.6	-7.7	92.3
		2.5	2.3	0.1	4.3	-4.9	95.1

		0.3	0.3	0.0	1.1	10.7	110.7
		0.1	0.1	0.0	12.3	2.8	102.8
**Chry**	2.47	200.00	181.73	18.01	9.91	-9.14	90.86
		66.67	64.11	4.64	7.23	-3.84	96.16
		22.22	24.40	1.79	7.34	9.82	109.82
		7.41	7.01	0.27	3.85	-5.39	94.61
		2.47	2.52	0.13	5.01	2.10	102.10
**B(b)F**	2.47	200.0	181.5	14.6	8.0	-9.2	90.8
		66.7	62.6	4.0	6.4	-6.1	93.9
		22.2	23.7	1.0	4.4	6.7	106.7
		7.4	6.9	0.2	3.4	-7.2	92.8
		2.5	2.5	0.1	5.4	1.6	101.6
**B(a)P**	0.09	200.0	185.8	12.0	6.4	-7.1	92.9
		66.7	64.5	3.1	4.8	-3.2	96.8
		22.2	24.2	1.6	6.6	8.9	108.9
		7.4	6.8	0.3	3.9	-7.6	92.4
		2.5	2.4	0.2	9.4	-1.3	98.7
**IndP**	2.47	200.0	187.1	9.9	5.3	-6.5	93.5
		66.7	66.0	1.4	2.2	-1.0	99.0
		22.2	26.4	2.5	9.4	18.9	118.9
		7.4	7.0	0.2	3.4	-5.8	94.2
		2.5	2.5	0.1	3.6	-0.4	99.6
		0.8	1.0	0.1	13.1	15.5	115.5
**D(ah)A**	0.09	200.0	195.2	4.8	2.5	-2.4	97.6
		66.7	66.0	1.6	2.4	-1.0	99.0
		7.4	7.2	0.3	4.4	-2.5	97.5
		2.5	2.6	0.1	3.7	5.7	105.7
		0.3	0.3	0.0	3.3	-5.1	94.9

		0.1	0.1	0.0	5.4	4.0	104.0
**B(ghi)P**	0.27	200.0	201.0	6.7	3.3	0.5	100.5
		66.7	68.4	2.2	3.2	2.6	102.6
		7.4	7.0	0.3	4.6	-5.2	94.8
		2.5	2.4	0.1	4.7	-2.0	98.0
		0.8	0.9	0.0	2.9	10.4	110.4

[a]An aqueous solution of nicotelline, nicotine, TSNAs and PAHs containing the specified amounts was added to 200 mg powdered, dried clay soil. Samples were extracted and analyzed as described for the SHD in the experimental section. For all compounds, results are based on the mean of four replicates. LOQ: Limit of quantitation (ng/g); SD: standard deviation; RSD: relative standard deviation.

Supplementary Table 3. Equations for typical standard curves

Pollutant	Concentration range (ng/g)	Slope[a]	Intercept	Correlation coefficient (r^2)
**Nicotine**	0-200,000	0.191056	0.00698	0.9986
**Nicotelline**	0-200	0.35566	0.00184	0.9982
**NNK**	0-200	0.206839	-0.00759	0.9992
**NNN**		0.144304	-0.00012	1.0000
**NAT**		0.133022	1.99774	0.9993
**NAB**		0.786727	0.145523	0.9976
**Ph**	0-10,000	0.227839	0.062935	0.9975
**An**		0.016556	0.004256	0.9903
**Fluo**		0.037721	0.054607	0.9979
**Pyr**		0.035267	0.038995	0.9991
**BaA**		0.035974	0.013190	0.9939
**Chry**		0.032483	0.005760	0.9940
**BbF**		0.034311	0.003298	0.9938
**BaP**		0.025556	0.012023	0.9998

**IndP**		0.030388	0.012978	0.9998
**DahA**		0.047156	-0.006188	0.9992
**BghiP**		0.037242	0.013107	0.9993

[a]Equations were determined by linear regression: response ratio = a × (amount ratio) + intercept.

Journal of Environmental Exposure Assessment

Supplementary Table 4. Descriptive statistics for all pollutants (in ng/g) of outdoor deposited house dust collected in Malta

**Pollutant**	**Valid *n***	**Detection frequency (%)**	**Mean**	**SD**	**Min**	**Max (ng/g)**	**Q1**	**Q2**	**Q3**
**Nicotine**	16	80	3692	11428	75	46,364	213	580	1414
**Nicotelline**	20	100	2	4	0	17	0	1	2
**NNK**	20	100	0.5	0.9	0.0	2.9	0.1	0.2	0.3
**NNN**	20	100	0.4	1.0	0.0	4.3	0.0	0.1	0.2
**NAT**	20	100	0.2	0.4	0.0	2.1	0.0	0.0	0.1
**NAB**	20	100	0.2	0.2	0.0	0.7	0.1	0.1	0.3
**Σ TSNA**	20	100	1.3	2.3	0.1	9.9	0.2	0.5	0.9
**Ph**	20	100	135	81	24	326	82	110	180
**An**	20	100	13	10	1	37	6	10	21
**Fluo**	20	100	178	124	32	506	83	152	246
**Pyr**	18	90	171	110	33	463	104	135	207

© The Author(s) 2021. Open Access This article is licensed under a Creative Commons Attribution 4.0 International License (https://creativecommons.org/licenses/by/4.0/), which permits unrestricted use, sharing, adaptation, distribution and reproduction in any medium or

format, for any purpose, even commercially, as long as you give appropriate credit to the original author(s) and the source, provide a link to the Creative Commons license, and indicate if changes were made.

**BaA**	20	100	57	37	5	146	31	54	84
**Chry**	20	100	110	73	15	308	56	97	156
**BbF**	20	100	87	62	16	276	43	85	114
**BaP**	20	100	78	50	11	202	44	77	101
**IndP**	20	100	74	49	13	196	32	70	100
**DahA**	20	100	18	14	2	44	7	17	27
**BghiP**	20	100	92	61	14	257	40	90	106
**Σ PAH**	20	100	1074	717	133	3034	599	1009	1265

Q1,Q2, Q3 are the 25th, 50th and 75th percentiles respectively.

Supplementary Table 5. Mann Whitney U test for all pollutants in SHD to check statistical difference across smoking and non-smoking houses

**Test statistics[a,b]**																			
	Nicotine	Nicotelline	NNK	NNN	NAB	NAT	∑TSNA	Ph	An	Fluo	Pyr	BaA	Chry	BbF	BaP	IndP	DahA	BghiP	∑PAH
Mann-Whitney *U*	8.000	128.000	132.000	88.000	131.500	99.500	112.000	121.000	157.000	51.000	46.000	65.000	45.000	48.000	92.000	54.000	81.000	101.000	74.000
Wilcoxo	74.0	869.0	873.	829.	872.	840.	853.	226.	262.	106.	101.	143.	111.	103.	158.	90.	172.	192.	179.

n W	00	00	000	000	500	500	000	000	000	000	000	000	000	000	000	000	000	000	000
Z	-3.310	-3.334	-3.259	-4.136	-3.268	-3.916	-3.637	-2.243	-1.335	-3.023	-3.184	-3.202	-3.531	-3.120	-2.045	-2.287	-3.015	-2.585	-3.506
Asymp. Sig. (2-tailed)	< 0.001	< 0.001	0.001	< 0.001	0.001	< 0.001	< 0.001	0.025	0.182	0.002	0.001	0.001	< 0.001	0.002	0.041	0.022	0.003	0.010	< 0.001
Exact Sig. [2*(1-tailed Sig.)]	< 0.001[b]									0.002[b]	< 0.001[b]	< 0.001[b]	< 0.001[b]	0.001[b]	0.041[b]	0.021[b]	0.002[b]		

[a]Grouping variable: smoker; [b]not corrected for ties.

Supplementary Table 6. Kruskal-Wallis H test for all pollutants in SHD to check statistical difference across the different districts in Malta

**Test statistics[a,b]**																			
	Nicoti	Nicotell	NN	NN	NA	NA	∑TS	Ph	An	Flu	Pyr	Ba	Chr	Bb	BaP	Ind	Dah	Bgh	∑PA

 Surname *et al. Metab Target Organ Damage* Year;Volume:Number | http://dx.doi.org/10.20517/mtod.xxxx.xx

	ne	ine	K	N	T	B	NA			o		A	y	F		P	A	iP	H
Kruskal-Wallis H	3.941	4.262	5.995	4.010	.933	.992	5.579	4.150	1.790	5.411	3.922	3.163	3.825	4.816	4.596	3.565	3.392	4.558	4.068
df	4	4	4	4	4	4	4	4	4	4	4	4	4	4	4	4	4	4	4
Asymp. Sig.	0.414	0.372	0.200	0.405	0.920	0.911	0.233	0.386	0.774	0.248	0.417	0.531	0.430	0.307	0.331	0.468	0.495	0.336	0.397

[a]Kruskal Wallis test; [b]Grouping variable: district.

Supplementary Table 7. Spearman rho correlation plot for nicotine, nicotelline, TSNA and PAHs

Correlations

			Nicotelline	Nicotine	NNK	NNN	NAT	NAB	TotalTSNA	Ph	An	Fluo	Pyr	BaA	Chry	BbF	BaP	IndP	DahA	BghIP	TotalPAH
Spearman's rho	Nicotelline	Correlation Coefficient	1.000																		
		Sig. (2-tailed)																			
		N	54																		
	Nicotine	Correlation Coefficient	.697**	1.000																	
		Sig. (2-tailed)	0.000																		
		N	21	21																	
	NNK	Correlation Coefficient	.905**	.709**	1.000																
		Sig. (2-tailed)	0.000	0.000																	
		N	54	21	54																
	NNN	Correlation Coefficient	.819**	.760**	.847**	1.000															
		Sig. (2-tailed)	0.000	0.000	0.000																
		N	54	21	54	54															
	NAT	Correlation Coefficient	.605**	.760**	.616**	.790**	1.000														
		Sig. (2-tailed)	0.000	0.000	0.000	0.000															
		N	54	21	54	54	54														
	NAB	Correlation Coefficient	.459**	.612**	.609**	.572**	.530**	1.000													
		Sig. (2-tailed)	0.000	0.003	0.000	0.000	0.000														
		N	54	21	54	54	54	54													
	TotalTSNA	Correlation Coefficient	.883**	.765**	.972**	.903**	.717**	.708**	1.000												
		Sig. (2-tailed)	0.000	0.000	0.000	0.000	0.000	0.000													
		N	54	21	54	54	54	54	54												
	Ph	Correlation Coefficient	-0.073	-0.187	0.026	-0.076	-0.210	-0.048	-0.039	1.000											
		Sig. (2-tailed)	0.637	0.458	0.867	0.622	0.171	0.758	0.802												
		N	44	18	44	44	44	44	44	44											
	An	Correlation Coefficient	0.117	-0.362	0.099	-0.019	-0.127	0.058	0.071	.625**	1.000										
		Sig. (2-tailed)	0.451	0.140	0.522	0.902	0.412	0.708	0.647	0.000											
		N	44	18	44	44	44	44	44	44	44										
	Fluo	Correlation Coefficient	-0.143	-.532*	-0.112	-0.253	-0.271	-0.231	-0.160	.845**	.589**	1.000									
		Sig. (2-tailed)	0.385	0.034	0.496	0.121	0.095	0.157	0.332	0.000	0.000										
		N	39	16	39	39	39	39	39	39	39	39									
	Pyr	Correlation Coefficient	-0.070	-0.331	-0.028	-0.241	-0.253	-0.173	-0.095	.806**	.702**	.936**	1.000								
		Sig. (2-tailed)	0.672	0.195	0.867	0.140	0.121	0.293	0.565	0.000	0.000	0.000									
		N	39	17	39	39	39	39	39	39	39	38	39								
	BaA	Correlation Coefficient	-0.096	-.558*	-0.131	-.309*	-.307*	-0.174	-0.174	.769**	.737**	.922**	.928**	1.000							
		Sig. (2-tailed)	0.544	0.016	0.408	0.046	0.048	0.269	0.271	0.000	0.000	0.000	0.000								
		N	42	18	42	42	42	42	42	42	42	39	39	42							
	Chry	Correlation Coefficient	-0.085	-.496*	-0.063	-0.287	-.328*	-0.176	-0.132	.777**	.672**	.922**	.973**	.949**	1.000						
		Sig. (2-tailed)	0.598	0.036	0.697	0.068	0.036	0.271	0.412	0.000	0.000	0.000	0.000	0.000							
		N	41	18	41	41	41	41	41	41	41	39	39	41	41						
	BbF	Correlation Coefficient	-0.135	-.521*	-0.146	-0.298	-.325*	-0.236	-0.195	.745**	.616**	.928**	.933**	.942**	.956**	1.000					
		Sig. (2-tailed)	0.413	0.039	0.374	0.065	0.044	0.149	0.233	0.000	0.000	0.000	0.000	0.000	0.000						
		N	39	16	39	39	39	39	39	39	39	39	38	39	39	39					
	BaP	Correlation Coefficient	-0.102	-0.224	-0.127	-0.205	-0.204	-0.180	-0.156	.702**	.773**	.776**	.852**	.871**	.813**	.833**	1.000				
		Sig. (2-tailed)	0.530	0.405	0.434	0.204	0.206	0.265	0.336	0.000	0.000	0.000	0.000	0.000	0.000	0.000					
		N	40	16	40	40	40	40	40	40	40	39	38	39	39	39	40				
	IndP	Correlation Coefficient	-0.069	-0.339	-0.093	-0.198	-0.189	-0.091	-0.116	.752**	.651**	.926**	.889**	.940**	.910**	.959**	.846**	1.000			
		Sig. (2-tailed)	0.684	0.216	0.585	0.240	0.262	0.592	0.494	0.000	0.000	0.000	0.000	0.000	0.000	0.000	0.000				
		N	37	15	37	37	37	37	37	37	37	37	36	37	37	37	37	37			
	DahA	Correlation Coefficient	-0.170	-.527*	-0.180	-.304*	-.322*	-0.134	-0.205	.667**	.676**	.873**	.863**	.904**	.899**	.913**	.906**	.951**	1.000		
		Sig. (2-tailed)	0.277	0.025	0.249	0.048	0.035	0.393	0.186	0.000	0.000	0.000	0.000	0.000	0.000	0.000	0.000	0.000			
		N	43	18	43	43	43	43	43	43	43	39	39	42	41	39	40	37	43		
	BghIP	Correlation Coefficient	-0.080	-0.360	-0.102	-0.212	-0.239	-0.055	-0.115	.701**	.640**	.894**	.903**	.893**	.913**	.936**	.903**	.965**	.957**	1.000	
		Sig. (2-tailed)	0.607	0.142	0.509	0.166	0.119	0.721	0.458	0.000	0.000	0.000	0.000	0.000	0.000	0.000	0.000	0.000	0.000		
		N	44	18	44	44	44	44	44	43	43	39	39	42	41	39	40	37	43	44	
	TotalPAH	Correlation Coefficient	-0.115	-0.389	-0.085	-0.248	-.300*	-0.085	-0.134	.851**	.660**	.939**	.973**	.950**	.971**	.946**	.888**	.919**	.873**	.905**	1.000
		Sig. (2-tailed)	0.452	0.111	0.579	0.101	0.045	0.579	0.382	0.000	0.000	0.000	0.000	0.000	0.000	0.000	0.000	0.000	0.000	0.000	
		N	45	18	45	45	45	45	45	44	44	39	39	42	41	39	40	37	43	44	45

**. Correlation is significant at the 0.01 level (2-tailed).

*. Correlation is significant at the 0.05 level (2-tailed).

Supplementary Table 8. Nicotine, ∑ TSNA and ∑ PAH concentrations (in ng/g) and % load of pollutant in indoor/outdoor and smoker/non-smokers' SHD

Place	N	Nicotine (%)	∑ TSNA (%)	∑ PAH (%)
**Indoor**	54	18246<br>**85.1**	26<br>**0.1**	3169<br>**14.8**
**Outdoor**	20	3692<br>**77.4**	1.3<br>**0.1**	1074<br>**22.5**
**Indoor-smokers**	16	37366<br>**97.9**	40<br>**0.1**	762<br>**2.0**
**Indoor-non-smokers**	30	864<br>**16.8**	20<br>**0.4**	4256<br>**82.8**

All concentrations are mean values (in ng/g).
